# Hydrochemical characteristics of surface waters and their relationships to the Kashin–Beck Disease in Longzi County, Tibet

**DOI:** 10.1038/s41598-022-11463-7

**Published:** 2022-05-12

**Authors:** Xinjie Zha, Yuan Tian, Jianyu Xiao, Chengqun Yu

**Affiliations:** 1 Xi’an University of Finance and Economics, Xi’an, 710100 China; 2 Key Laboratory of Ecosystem Network Observation and Modelling, Institute of Geographic Sciences and Natural Resources Research, Chinese Academy of Sciences, Beijing, 100101 China; 3 University of Chinese Academy of Sciences, Beijing, 100049 China

**Keywords:** Environmental chemistry, Element cycles, Environmental impact

## Abstract

Although previous studies have been reported between the Kashin–Beck Disease (KBD) epidemic and the hydrochemical characteristics of surface waters, the etiology of the disease remains unclear. In the present study, we comprehensively investigated the relationship between the KBD and the hydrochemical characteristics of surface waters in Longzi County. Results show that, the pH (mean = 7.27 ± 0.30), total hardness (TH, mean = 57.08 ± 45.74 mg L^−1^), total dissolved solids (TDS, mean = 67.56 ± 44.00 mg L^−1^) and oxidation–reduction potential (ORP, mean = 84.11 ± 23.55 mV) of surface waters in KBD endemic areas are lower than those in the non-KBD endemic areas (means of pH = 7.49 ± 0.30; TH = 262.06 ± 123.29 mg L^−1^; TDS = 253.25 ± 100.39 mg L^−1^; ORP = 215.90 ± 55.99 mV). These results suggest that long-term consumption of low TDS, essential trace elements (e.g., nickel, cobalt, iron, selenium, zinc, molybdenum, and iodine) deficient, and potential toxic elements (e.g., arsenic) enriched waters by humans likely causes the KBD. Environmental factors such as the geology and geomorphology may produce biogeochemical imbalance, geomorphic, vegetation types and local climatic conditions may have significant impact on food fungi toxin poisoning and water organic compound poisoning, and these also impact the KBD occurrence.

## Introduction

The hydrosphere around inhabited areas directly or indirectly affects human^1^. As an important intake source of essential trace elements (ETEs) and potentially toxic elements (PTEs), drinking water are closely associated with human health^2–4^. Geological, geographical, ecological, and environmental conditions account for differences in physicochemical characteristics of water in nature^5–8^. However, these characteristics are mainly controlled by the baseline concentrations of elements that can be easily leached from rocks and soils through which water flows^8–10^. Several studies on the relationships between drinking water and diseases, such as cancer^11,12^, infections^13^, and organic disease^14^, especially endemic diseases^15,16^ are available.

**Figure 1:**
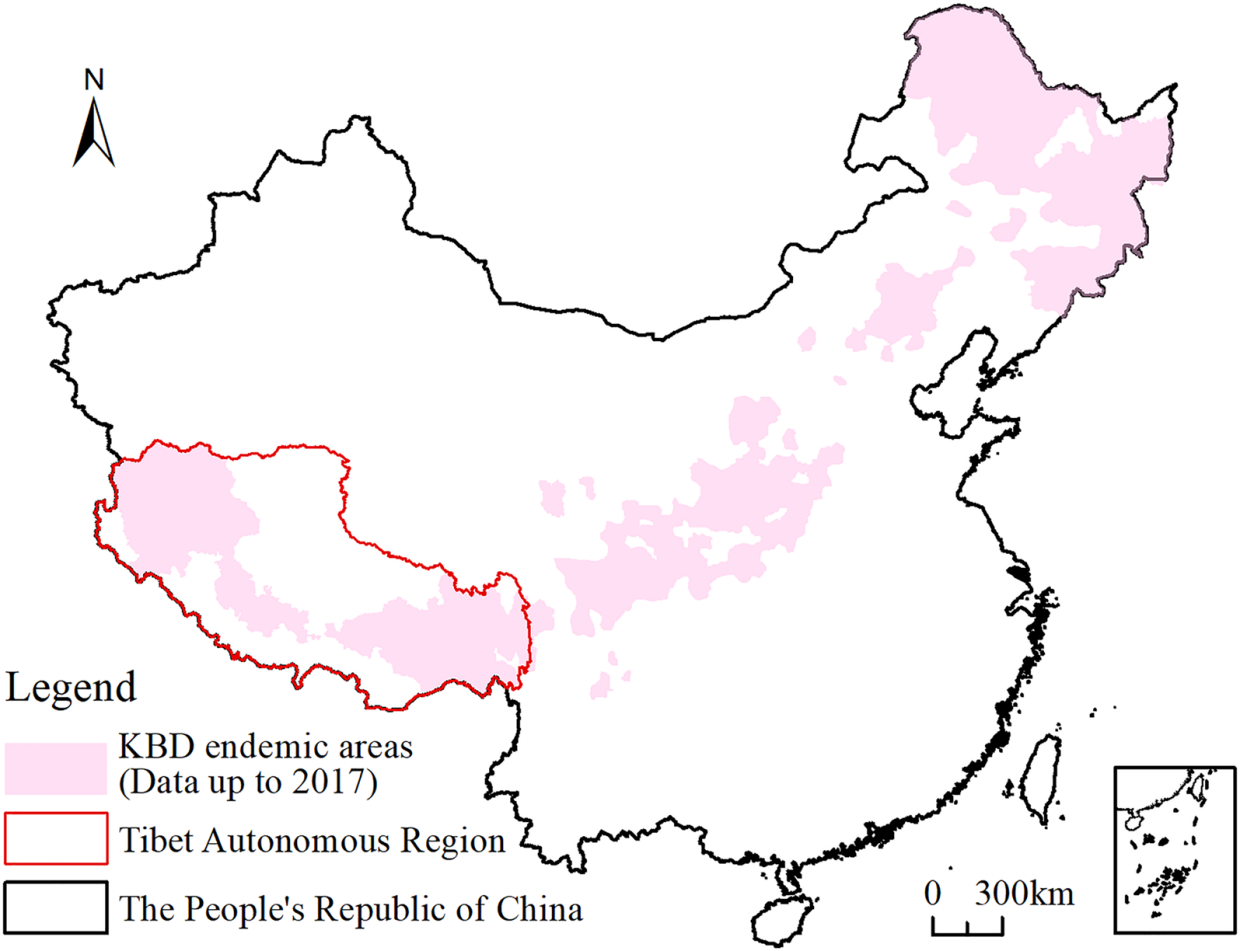
Distribution of sites that have been affected by KBD in China (data cited from Dinh et al.^28^; this figure was created with ArcGIS 10.8, URL: https://www.esri.com/en-us/arcgis/products/index).

The Kashin–Beck Disease (KBD) is a chronic, multisite, endemic osteochondropathy involving cartilage and growth plates. It mainly affects the growth and development of children including adolescents by causing necrosis, reshaping of joints, and shortening of extremities^17–20^. This disease has been reported from the Qinghai–Tibet Plateau in the southwest to the Heilongjiang Province in the northeast, thereby producing a belt-shaped occurrence in China^21,22^ (Fig. 1). Although the KBD has essentially controlled in China at present, however, new clinical cases still emerge, especially in endemic zones in Tibet. This severely impacts the quality of life and physical health of local residents as well as retards the economic and social development of endemic areas^23–26^. Approximately 9122 people (clinical degree class *I* and above) in 178 townships of 54 counties (73% of counties) in Tibet are facing adversity linked to the KBD^27^.

Although several studies on the etiology of the KBD exist, specifics on this, such as the pathogenesis are generally lacking. Presently, the following etiological hypotheses for the KBD have been proposed: (1) endemic deficiency of trace elements in local diets^29,30^, (2) significant mycotoxin contamination of grains by fungi^31,32^, and (3) excessive humic acid concentrations in drinking water^33,34^. Field and epidemiological/experimental studies support each of these theories, but none can adequately explain the pathogenesis and etiology of the KBD. In many studies, the KBD is attributed to multiple causes, with a deficiency in selenium (Se) representing the most established theory^23,26,29,35–37^. A number of studies have been published inspired by this Se-deficiency theory, attempting to link KBD to various environmental media, notably, hydrochemical characteristics^30,38–46^.

In the Qinghai–Tibet Plateau, existing studies on the hydrochemical characteristics of water and the correlation with the KBD mainly focused on the northwestern Sichuan^36,40,47^, Qinghai^39^, some counties in central Tibet^38,48,49^, and Qamdo City in the northeast of Tibet^26,50^, without any systematic study in the south of Tibet. In addition, existing studies on the relationships between hydrochemical characteristics of surface waters and KBD in Tibet mostly focus on few selected trace elements in some villages, lacking a comprehensive study on a county-wide scale. Based on aforementioned considerations, in order to further study the environmental causal factors of the KBD, this work explored the issue in a larger scope by: (a) investigating comprehensively the diverse environmental impact factors of hydrochemical characteristics in the whole Longzi County instead of focusing on a few critical elements in few regions; (b) how environmental factors affect the prevalence of KBD by controlling the hydrochemical characteristics of surface waters. The results of this cross-sectional study contribute to provide a basis on targeted prevention and intervention of KBD for local disease control and prevention departments, and hope to bring some new enlightenment on the pathogenesis of KBD.

## Results

### pH, TH, TDS and ORP

In general, surface waters in Longzi County are neutral to slightly alkaline because pH values range between 6.61 and 8.07 (mean = 7.39). The maximum pH value of 8.07 was obtained from a location near the tributary of the Longzi Xiongqu River in Liemai, while the minimum value of 6.61 was produced by a mountain spring sample from the Yumai. The measured pH values shown in Table 1 produce a range similar to the 6.87–8.14 reported by Tian, et al.^6^ for waters in the southeast border of Tibet. In the present study, pH values generally decreased from upstream to near neutral downstream, and values for samples from townships near the central area of the Longzi Xiongqu River including Liemai (7.50), Jiayu (7.55), and Zhunba (7.59) are higher than those upstream, such as Rerong (7.39), Ridang (7.27), and Longzi (7.38). Samples collected from townships affected by the KBD in Longzi County, such as Yumai and Zhari, exhibit relatively low pH values ranging between 6.61 and 7.91, with a mean value of 7.27 (Table 1). The ORP value range between 42 and 393 mV (mean = 204.89 mV). It also can be seen in Table 1, ORP values generally decreased from non-KBD areas (mean = 215.90 mV) to KBD areas (mean = 84.11 mV). Low water pH and ORP is usually associated with a reducing environment, in which humic acids and other organic substances accumulate; such an environment is conducive for the incidence of the KBD^51^.

**Table 1:** Summary of pH, TH, TDS and ORP data for surface water samples from different townships in the Longzi County.

Township	Number of samples	pH			TH (mg L^−1^)			TDS (mg L^−1^)			ORP (mV)		
		Mean	Range	SD^a^	Mean	Range	SD^a^	Mean	Range	SD^a^	Mean	Range	SD^a^
Longzi	3	7.38	6.63–7.78	0.57	210.38	164.3–260.2	48.09	216.94	170.0–267.0	48.58	246.67	210–272	32.52
Ridang	11	7.27	6.73–7.66	0.28	141.03	35.2–321.5	76.43	172.54	46.0–473.0	133.77	287.18	228–393	44.03
Liemai	6	7.50	7.06–8.07	0.35	438.42	340.6–510.8	72.17	372.06	305.0–447.0	57.35	205.67	187–243	20.41
Rerong	4	7.39	7.01–7.72	0.36	333.5	195.8–528.3	141.73	314.43	175.7–454.0	114.35	266.00	248–302	24.43
San’anqulin	8	7.30	6.93–7.77	0.25	213.06	92.5–293.5	59.86	217.88	96.0–277.0	58.23	166.88	110–228	37.19
Zhunba	7	7.59	7.44–7.75	0.12	225.25	96.2–365.3	99.12	234.86	115.0–337.0	83.69	158.86	101–266	54.17
Xuesa	9	7.55	6.96–8.04	0.37	308.36	65.1–489.9	141.25	280.59	80.0–387.7	110.09	213.33	166–280	31.95
Zhari	19	7.32	6.81–7.91	0.26	54.03	15.6–86.9	22.69	64.21	18.0–103.0	24.77	90.89	43–133	24.66
Yumai	17	7.22	6.61–7.83	0.34	60.49	9.0–281.4	63.05	71.29	14.0–266.0	59.29	76.53	42–112	20.31
Jiayu	12	7.55	7.25–8.03	0.20	315.67	181.5–484.3	110.98	265.34	184.0–364.0	69.46	164.13	137–217	24.42
Douyu	8	7.75	7.44–7.96	0.20	228.56	143.8–346	56.43	260.75	172.0–371.0	58.00	212.83	161–289	34.26
Non-KBD areas	68	7.49	6.88–8.07	0.30	262.06	35.2–528.3	123.29	253.25	46.0–473.0	100.39	215.90	151–343	55.99
KBD areas	36	7.27	6.61–7.91	0.30	57.08	9.0–281.4	45.74	67.56	14.0–266.0	44.00	84.11	42–133	23.55
Whole county	104	7.41	6.61–8.07	0.32	191.1	9.0–528.3	142.13	188.97	14.0–473.0	122.86	204.89	42–393	77.32

^a^Standard deviation (SD).

The TH and TDS are important indicators of the quality of drinking water. Based on the TH values, water in the area can be divided into soft (< 150 mg L^−1^), slightly hard (150–300 mg L^−1^), hard (300–450 mg L^−1^) and extremely hard water (> 450 mg L^−1^). The TH values for surface waters from the nine non-KBD townships range between 35.2 and 528.3 mg L^−1^, while mean values vary from 141.0 to 438.4 mg L^−1^, and these indicate that the waters are soft to hard (Table 1). However, surface waters in both towns affected by the KBD are soft, with TH values ranging between 9.0 and 281.4 mg L^−1^, and mean values varying from 54.0 to 60.5 mg L^−1^. As presented in Table 1, the TDS values for surface waters in the nine non-KBD townships vary between 46.0 and 473.0 mg L^−1^ (mean = 253.3 mg L^−1^), while those for the two KBD townships range between 14.0 and 266.0 mg L^−1^ (67.6 mg L^−1^).

### Hydrochemical characteristics

The concentrations of four major cations including sodium (Na^+^), magnesium (Mg^2+^), potassium (K^+^) and calcium (Ca^2+^), and four major anions including chlorine (Cl^−^), carbonate (CO_3_^2−^), bicarbonate (HCO_3_^−^) and sulfate (SO_4_^2−^) determine the hydrochemical characteristics of natural waters. The representation of these ions using a Piper diagram provides insights into physical and chemical processes controlling water chemistry^5^. Therefore, this methodology was employed in the present study for evaluation and classification of the surface waters.

**Figure 2:**
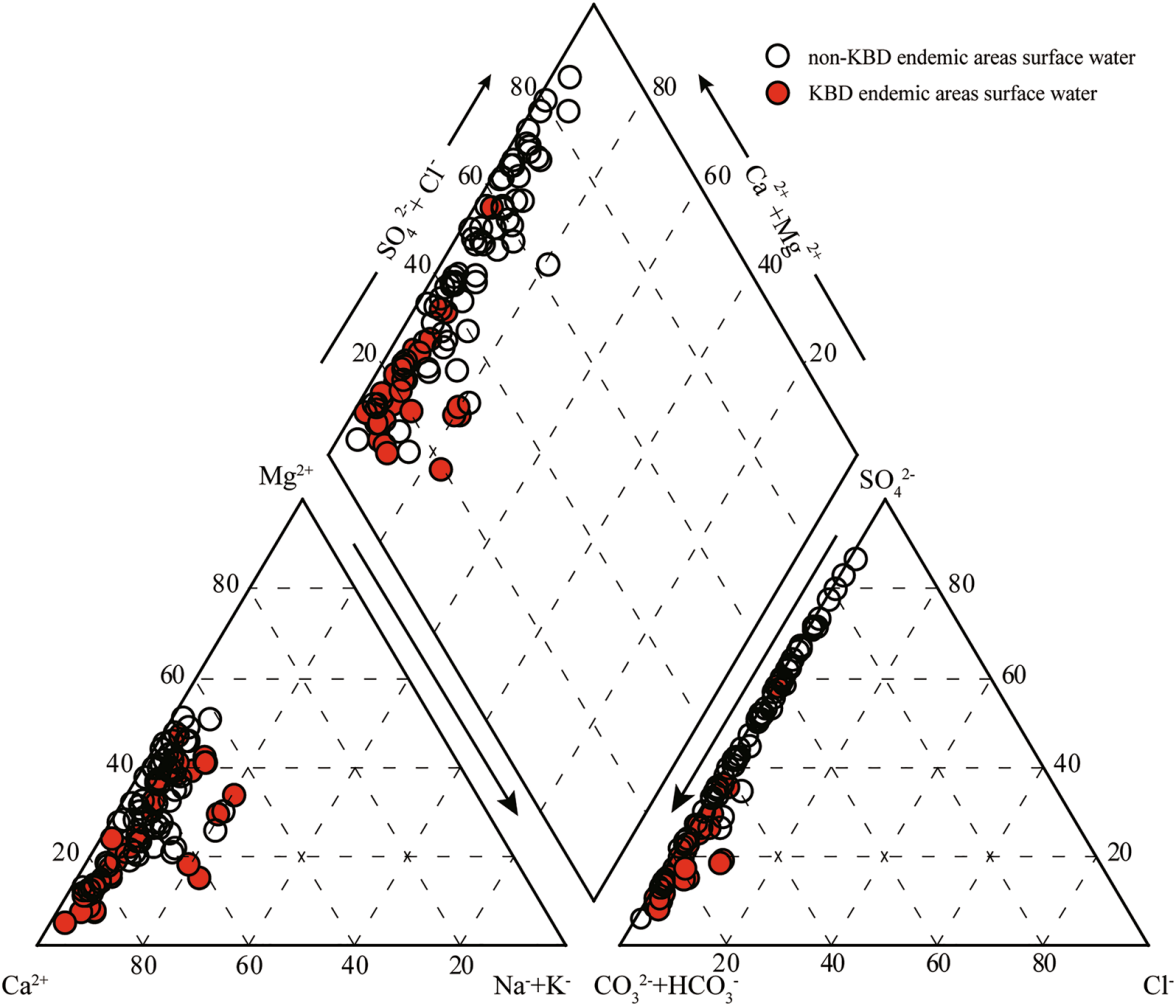
Piper diagram^5^ showing the characteristics of surface waters in the Longzi County.

As depicted in Fig. 2, HCO_3_^−^ and SO_4_^2−^ are the principal anions (approximately 57% and 42%, respectively) in surface waters of the non-KBD endemic areas in the west of the Longzi County, while the alkaline earth metal ions Ca^2+^ and Mg^2+^ are the primary cations (70% and 22%, respectively). Therefore, Ca–Mg–SO_4_–HCO_3_ and Ca–Mg–HCO_3_–SO_4_ are main types in water of these areas. In contrast, in the KBD endemic areas, HCO_3_^−^ (approximately 82%) is the predominant anion, while SO_4_^2−^ (approximately 17%) is significantly lower. The alkaline earth metal ions Ca^2+^ (75%) and Mg^2+^ (15%) account for approximately 90% of cations, while the alkali metal ions K^+^ and Na^+^ are significantly low (< 10%). Therefore, the surface water of KBD endemic areas are dominated by low TDS waters with Ca–HCO_3_ and Ca–Mg–HCO_3_ as the principal hydrochemical species. Xiao^52^ reported negative correlations between the incidence of the KBD and Na^+^ and Mg^2+^ deficiencies in drinking water. In addition, Hu^43^ indicated that the consumption of low-salinity water with low Mg^2+^, Cl^−^, SO_4_^2−^, and HCO_3_^−^ is a major cause of KBD. Further, based on the analysis of major ions in waters in the Rangtang County, Wang and Xie^51^ suggested that most drinking water are acidic waters with low ion contents, especially for SO_4_^2−^, in KBD endemic areas. Thus, based on previous studies, chemical characteristics of waters in the townships of Yuma and Zhari are conducive for KBD occurrence.

**Figure 3:**
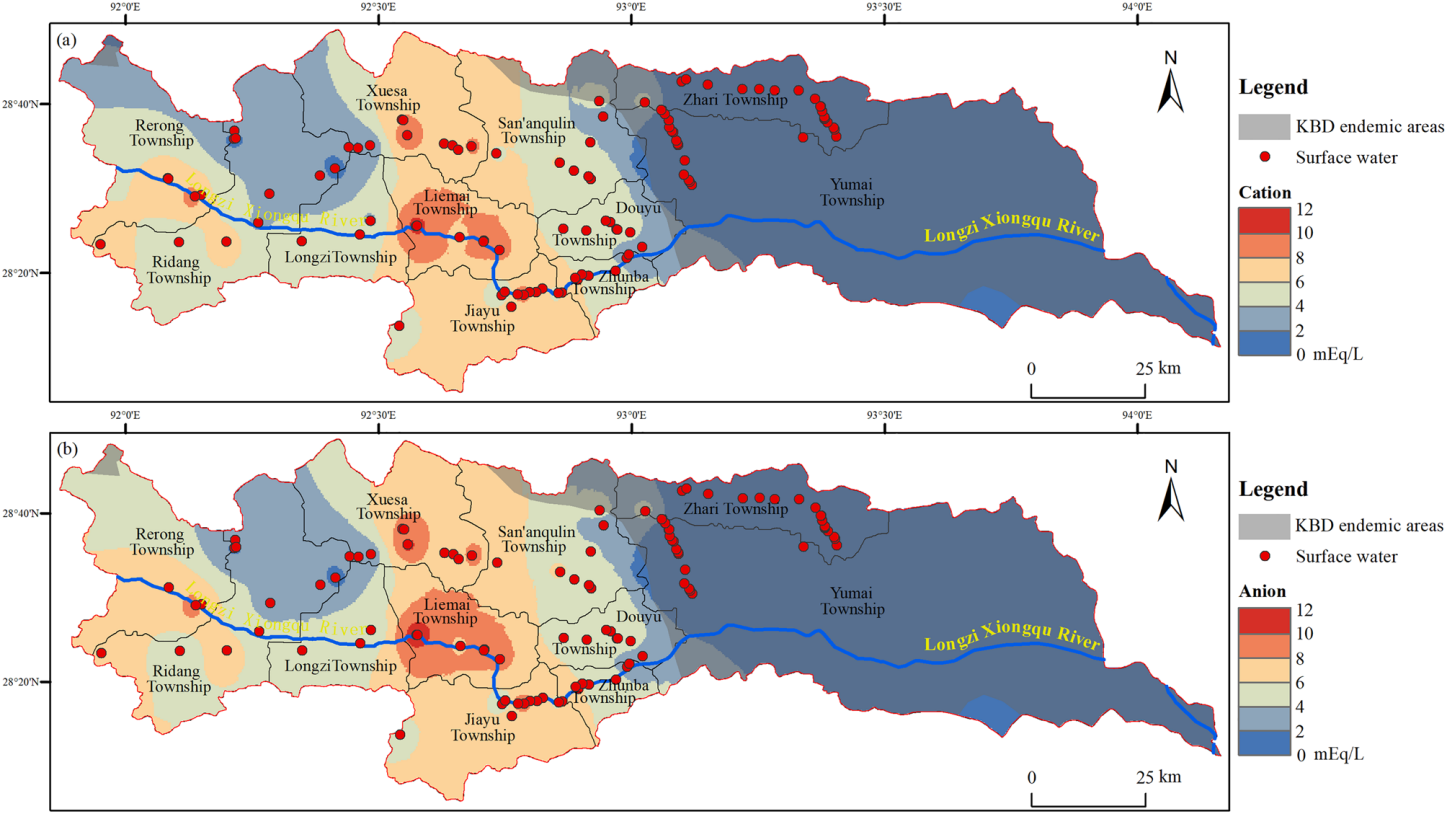
Maps showing distributions of the milliequivalent of (**a**) cations and (**b**) anions in surface waters in the Longzi County (this figure was created with ArcGIS 10.8, URL: https://www.esri.com/en-us/arcgis/products/index).

The measured total concentration of cations (Fig. 3a) and anions (Fig. 3b) are plotted as contours based on spatial interpolation. Obviously, both the cations and the anions exhibit lowest concentrations in the two townships in the east. Therefore, the spatial distribution of ions displays a relationship with the prevalence of the KBD in the Longzi County, which is consistent with previous studies in the Tibet Plateau^23,38,48^ and elsewhere in China^40,43,52^. These significant differences in the distribution of ions in the surface waters are attributed to many factors including the regional geology, local climate, and topography. As highlighted by the TDS and TH data, surface water TDS in the Longzi County is likely controlled by the Ca/Mg, carbonates/sulfates encountered. The interaction of rocks with flowing water causes dissolution of Ca/Mg carbonates and sulfates. The KBD endemic areas in Longzi County are on the south slope of the East Himalayas, which is characterized by a humid climate, lush vegetation, and waters with high organic contents. This regional setting can significantly limit water–rock interactions, thereby reducing the redox potential of the water system. Under such conditions, weathering is considerably decreased and this promotes low pH, salinity, and hardness, and these results are consistent with previous studies^40,51^. Other factors that affect the spatial distribution of ions in surface waters in the Longzi County, such as the geology, geography, and anthropogenic activities are examined subsequently.

### Possible sources of ions

**Figure 4:**
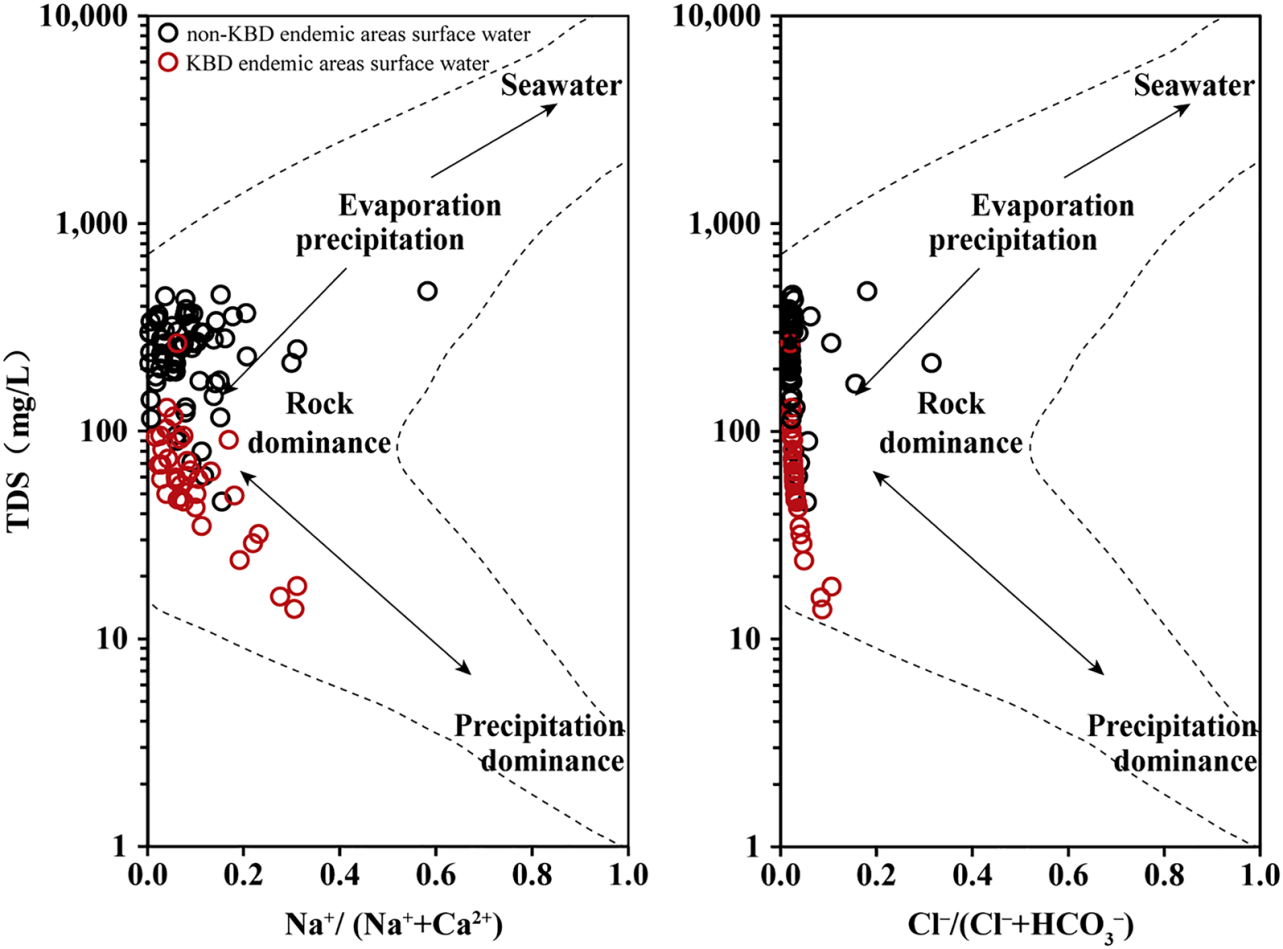
Gibbs boomerang envelope^7^ plot showing possible sources of ions in surface waters in the Longzi County.

In the Longzi County, surface water recharge is associated mainly with precipitation and melting of ice. The Gibbs boomerang envelope^7^ is a weight ratio simple plot of the TDS versus Na^+^/(Na^+^ + Ca^2+^) and the TDS versus Cl^−^/(Cl^−^ + HCO_3_^−^), which highlights the relative significance of evaporation, weathering, and precipitation on hydrochemical characteristics. According to the Gibbs boomerang envelope, rock weathering is the principal control on major hydrochemical components of surface waters in the Longzi County (Fig. 4). This observation is consistent with that from a previous study of rivers in the Qinghai–Tibet Plateau^6^. However, in the KBD endemic areas (Yumai and Zhari townships), the composition of surface waters is dominantly controlled by precipitation, while evaporation prevails in other townships (Fig. 4).

### Trace elements

Although trace elements in the natural environment are present in low concentrations in humans because these cannot be synthesized directly, elements such as iodine (I), copper (Cu), manganese (Mn), and zinc (Zn) are essential for health. Among trace elements, ETEs and PTEs, such as Mn, iron (Fe), cobalt (Co), Cu, and Zn have been associated with the KBD in many research^30,41,42,44–46^. In particular, the Se-deficiency theory has been advanced in several studies^50–52^. However, the relationship between the Se content and the incidence of the KBD suggests that although Se deficiency is a likely environmental factor, it does not represent the principal cause of the disease.

Data for trace elements and physicochemical parameters including aluminum (Al), silicon (Si), vanadium (V), Mn, Fe, Co, nickel (Ni), Cu, Zn, arsenic (As), Se, molybdenum (Mo), and I of surface waters in the non-KBD and KBD endemic areas in Longzi county, Tibet, are presented in Table 2. These elements were analyzed to evaluate their impact on the incidence of the KBD in the Longzi County. According to the results, Co, Fe, and Ni are normally distributed in the non-KBD endemic areas, while Fe, I, Mn, Mo, and V are normally distributed in townships affected by the KBD (> 0.1). According to data in Table 2, surface waters in the townships affected by the KBD exhibit lower concentrations for most trace elements compared to the non-KBD endemic areas. The Kruskal–Wallis (K–W) test results reveal significantly higher Co, Fe, I, Mo, Ni, Se, and Zn in the non-KBD endemic areas (*p* < 0.01), while the concentrations of As are significantly lower (*p* < 0.01).

**Table 2:** Summarized data for trace elements in surface waters from the non-KBD and KBD endemic areas in the Longzi County.

Parameters	Non-KBD							KBD							K–W
	Min^a^	Max^a^	Mean^a^	Median^a^	SD	CV^b^	K–S	Min^a^	Max^a^	Mean^a^	Median^a^	SD	CV^b^	K–S	*p* value
Al	0.37	20.37	5.34	3.17	5.09	13.88	0.00	0.93	22.22	6.19	4.13	5.58	6.00	0.01	0.21
Si	2.35	18.54	8.23	7.18	3.84	1.63	0.00	1.72	17.16	6.91	6.19	3.29	1.92	0.00	0.09
V	0.01	1.17	0.34	0.25	0.28	28.34	0.00	0.11	0.76	0.36	0.35	0.15	1.33	0.20	0.02
Mn	0.00	0.28	0.04	0.02	0.05	25.24	0.00	0.00	0.06	0.02	0.02	0.02	16.42	0.10	0.06
Fe	0.58	271.70	105.33	93.37	69.81	120.79	0.20	2.86	71.99	19.71	17.31	13.10	4.58	0.16	0.00
Co	0.02	0.18	0.06	0.06	0.03	1.80	0.12	0.01	0.44	0.04	0.02	0.08	15.94	0.00	0.00
Ni	0.02	2.47	1.15	1.06	0.52	25.76	0.20	0.11	0.84	0.32	0.29	0.17	1.58	0.01	0.00
Cu	0.00	0.64	0.16	0.10	0.17	165.49	0.00	0.02	0.44	0.15	0.14	0.10	5.43	0.00	0.37
Zn	0.03	5.49	1.03	0.55	1.18	34.84	0.00	0.01	3.57	0.47	0.18	0.89	177.33	0.00	0.00
As	0.01	1.55	0.29	0.18	0.33	41.57	0.01	0.17	1.49	0.58	0.49	0.32	1.95	0.02	0.00
Se	0.07	2.21	0.46	0.25	0.49	6.81	0.00	0.04	0.33	0.14	0.12	0.08	2.08	0.05	0.00
Mo	0.13	1.03	0.47	0.41	0.25	1.96	0.01	0.02	0.55	0.25	0.20	0.18	11.39	0.14	0.00
I	0.01	0.42	0.10	0.07	0.09	17.31	0.00	0.02	0.16	0.05	0.05	0.03	1.50	0.12	0.00

^a^Concentrations data are in μg L^−1^.

^b^Coefficient of variation (CV).

## Discussion

The environmental impact factors can help explain the etiology of KBD, because the hydrochemical characteristics of surface waters in the Longzi County is determined by diverse environmental impact factors including the geological (structural geology and lithology) and eco-geographical (geomorphic, vegetation types, and local climate) conditions, as well as human activities. In particular, the relationship between the KBD etiology and biogeochemical Se-deficiency has been the focus of many epidemiology and geochemical studies (for review, see the introduction section). Based on preliminary quantitative spatial analysis of the KBD risk factors in Tibet’s Qamdo City by our group, the pathogenesis and etiology of the disease are attributed to multiple interrelated factors. Moreover, these factors are synergistic and their interactions enhance the KBD prevalence^25^. In the present study, by comparing the hydrochemical characteristics in the non-KBD and KBD endemic areas, surface waters in the KBD endemic areas exhibit significant deficiencies in the TDS, TH, and ETEs, especially Fe, I, Mo, Mn, Ni, and Se (Table 2). This finding is consistent with previous studied on relationships between the KBD incidence and geochemical factors^21,38,40,43,50^. In addition to the results of surface waters trace elements concentrations presented in Table 2, PTEs, especially As, also display higher concentrations in the KBD endemic areas. Therefore, As is likely responsible for the etiology of the KBD, but additional quantitative evidence is required.

### Correlation and principal component analysis

**Figure 5:**
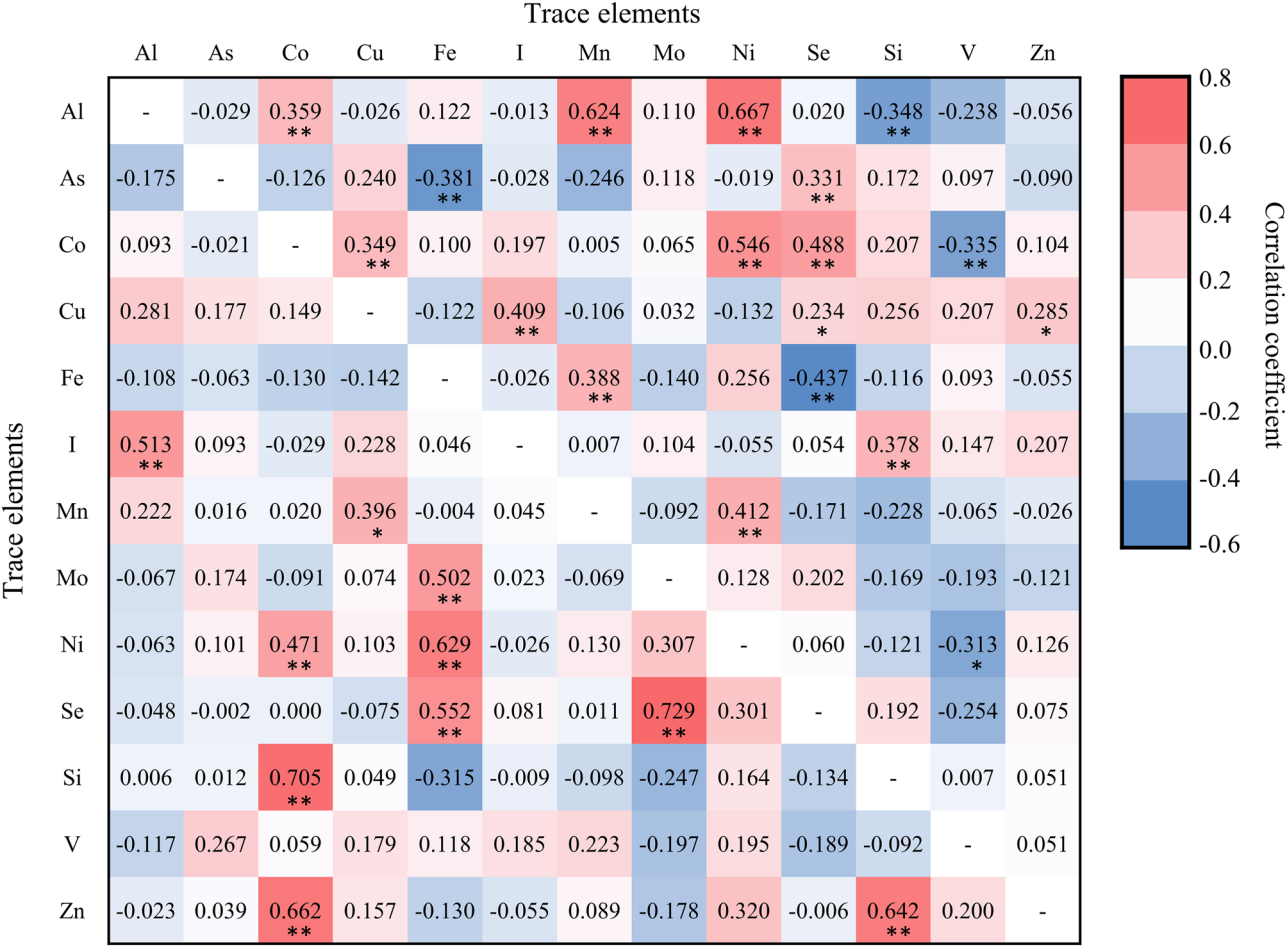
Correlation matrix for selected trace elements in surface waters from the non-KBD (upper right) and KBD (lower left) areas in the Longzi County. *Correlation is significant at *p* < 0.05 level; **correlation is significant at *p* < 0.01 level.

According to the Pearson correlation matrix in Fig. 5, relationships between trace elements in surface waters in the two areas differ, which indicate different sources or chemical behaviors^53^. For instance, in the non-KBD areas, strong positive correlations (*p* < 0.01) with coefficients varying between 0.359 and 0.667 for Al and Co, Mn, and Ni, and 0.349–0.546 for Co and Cu, Ni, and Se were obtained. However, in the KBD endemic townships, Al displays a significant correlation only with I (0.513), while Co is significantly correlated with Ni (0.471), Si (0.705), and Zn (0.662). These differences in correlations between trace elements in the KBD and non-KBD endemic areas are attributed to local conditions, which are examined subsequently.

**Figure 6:**
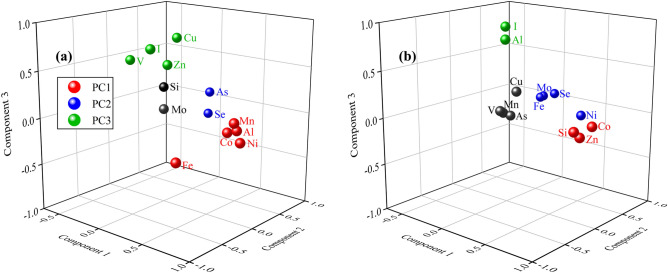
The first three PCs for trace elements in the (**a**) non-KBD and (**b**) KBD endemic areas in the Longzi County.

Principal component analysis (PCA) was conducted to explore possible sources of trace elements by extracting influential factors associated with the dataset^54^. Five and six principal components (PCs) were obtained for trace elements in surface waters from the non-KBD and KBD endemic areas, respectively, and the component matrixes and explained variance are presented in Supplementary Table S1. In the non-KBD endemic areas, PC1 explains 26.40% of the total variance, with high loadings for Ni, Mn, Al, Co, and Fe; PC2 involves As and Se, and it accounts for 14.5% of the total variance; PC3 explains 14.04% of the total variance, and this is associated primarily with Cu, I, V, and Z; PC4 represents 9.49% of the total variance, and this mainly attributed to Si and Mo. The five PCs with eigenvalues > 1 account for 73.23% of the total variance. In the KBD endemic areas, PC1 is dominated by Co, Si, and Zn, and this represents 21.21% of the total variance; PC2 is accounts for 19.20% of the total variance, and it dominated by Se, Mo, Fe, and Ni; PC3 and PC4 are mainly controlled corresponding by Al and I and by Cu and Mn, which represent 14.64% and 11.38% of the total variance, respectively. The six PCs with eigenvalues > 1 account for 82.65% of the total variance. The PCs associated with trace elements for both areas are shown in Fig. 6.

### Regional lithology and structural geology

Although the crustal abundance of elements is essentially stable, geological processes, such as tectonic movements and biogeochemical cycles create extreme differences in their spatial distribution. As noted in “Physical geography”, chemical components in natural waters in the Longzi County are mainly derived from the leaching and dissolution of rocks and soils produced by weathering of associated rocks (Fig. 4). Therefore, to adequately understand hydrochemical characteristics of surface waters in the Longzi County, the geology of the area must be carefully examined. Longzi County is in the central area of the east Himalayan–Tethys Orogenic Belt in the south of the Qinghai–Tibet Plateau. Therefore, it straddles the Himalayan Block and the Yarlung–Zangbo River Suture, with the Gangdese Landmass to its north. This area in the north margin of Gondwanaland has experienced extensive sedimentary–tectonic evolution since the Pan-African movement started, especially extension, contraction, and closure of the Tethys Ocean Basin since the Triassic. Intense collision between the Himalayan and Gangdese blocks produced large-scale extensional detachment, twisting, and strike-slip deformation, which explain the complicated sedimentation, magmatism, and metamorphism in the area.

**Figure 7:**
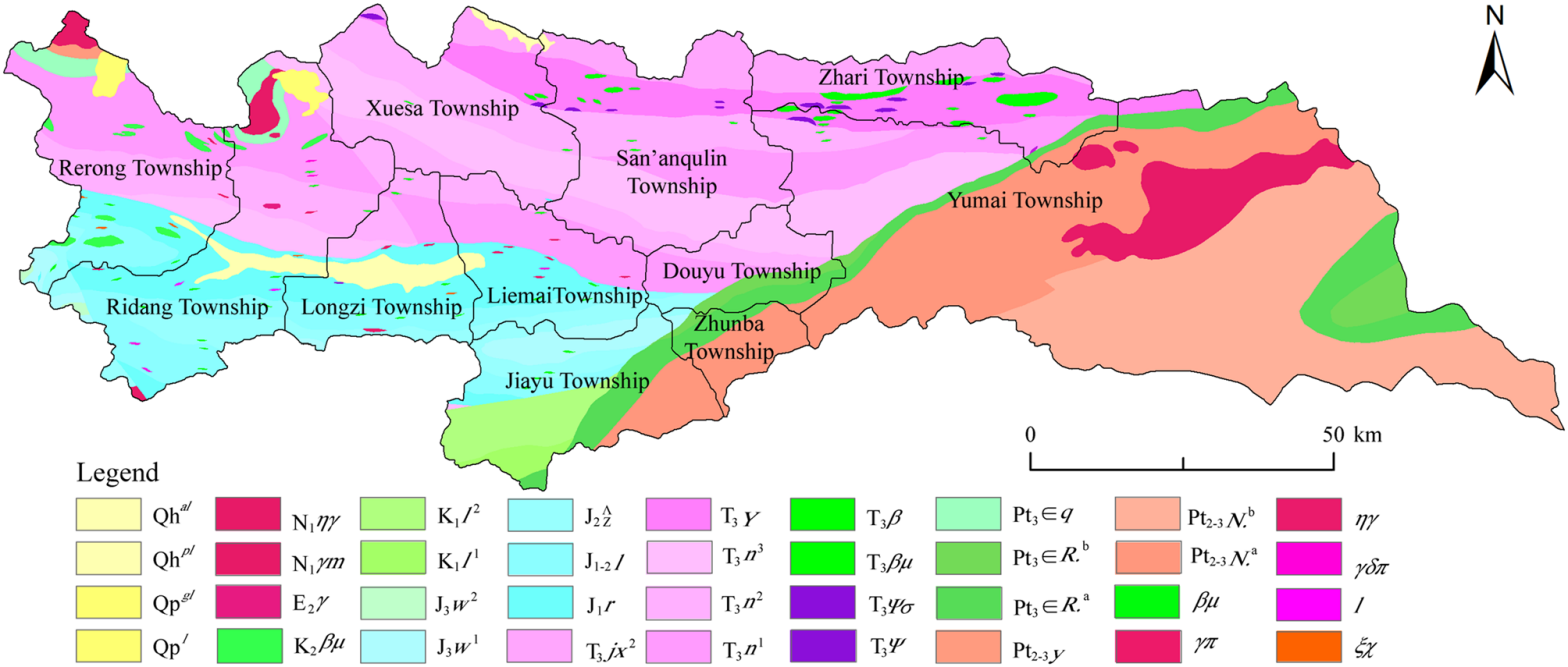
The geological map of the Longzi County (the strata distribution was cited from Digital Library of National Geological Archives of China, URL: http://www.ngac.org.cn; this figure was created with ArcGIS 10.8, URL: https://www.esri.com/en-us/arcgis/products/index).

Yumai and Zhari townships, which are in KBD endemic areas, belong to the third-level tectonic unit of the Yumen Tectonic Mélange in the Yarlung–Zangbo River Suture Zone. This tectonic unit is surrounded by the nearly E–W oriented Dengmu and Quzhula faults, and it is characterized by poorly-developed internal faults. Conversely, the nine townships in the non-KBD endemic areas fall within the Dala–San’anqulin and Zhegucuo–Ridang fold-and-thrust bundle third-level tectonic unit of the Kangma–Longzi Fold-and-thrust Belt. Structural deformation in the Kangma–Longzi Fold-and-thrust Belt produced mainly shallow facies and groups of nearly E, S, and NW trending faults. Field surveys reveal that large and medium fold structures in the non-KBD endemic areas are developed, and the rocks are characterized by more intense fracturing. These geological features are responsible for the abundance of fissure and pore water resources in the area, and these are rich in varied ions because of extensive interactions with rocks. These features also facilitate recharge by runoff and enhance the TDS of waters in the non-KBD endemic areas. Strata in the KBD endemic areas comprise mainly those of the Late Triassic Langjiexue Group and the Yumen Tectonic Mélange Formation (T_3_*Υ*), which contain rocks (T_3_*m*) and blocks (Fig. 7). These rocks, which belong mainly to the under-compensated sub-deep-sea basin sedimentary facies, comprise gray and dark gray silty sericite associated with a dark gray thin layered of siliceous rock. Most rock-forming minerals such as quartz and feldspar are insoluble in water. Blocks involve pyroxene-rich peridotites (T_3_Φσ), ultrabasic rocks (T_3_Σ), dunites (T_3_Φ), diabases (T_3_*βμ*), pillow lava basalts (T_3_*β*), etc. These rocks are also largely insoluble in water, and this partly accounts for the low TDS and TH of waters in the area. In the non-KBD endemic areas, strata belong mainly to the Late Triassic Nieru Formation (T_3_*n*) and Jurassic (J), which involve sub-stable to stable alternating marine and terrestrial facies as well as shallow sea shelf facies clastic sediments. Quaternary (Q*h*) strata are abundant in the Longzi River Basin of the Longzi and Ridang townships. In the non-KBD endemic areas, rocks with soluble components, such as limestone and clastic rocks including mudstones and sandstones are common, and carbonate rocks are abundant in the upper part of the Jurassic, which explains the high TDS and TH of the associated waters. Thus, hydrochemical characteristics of surface waters in the Longzi County are closely related to the geology. Further discussion and assessment on the influence of chemical compositions of bedrocks and overlying soils on the surface waters hydrochemical characteristics and its elements migration and transformation pattern are needed.

### Physical geography

The Himalayas is in the central part of the Longzi County, and these mountains, which are generally aligned from the NE to the SW, roughly separate the KBD from the non-KBD endemic areas. The KBD endemic areas are on the south slope of the Himalayas, while the non-KBD endemic areas are on the north slope. Zheng^55^ based on the spatial differentiation of thermal conditions, moisture regimes, and landform variations partitioned the Qinghai–Tibet Plateau into 10 natural zones and 28 physical districts. The KBD endemic areas are mainly on the plateau and lake basins around the north section of the Central Himalayas (IIC1 a), where annual precipitation varies between 200 and 400 mm, and the vegetation types on mostly alkaline soils are mainly shrubs and grasslands. Conversely, the non-KBD endemic areas are on the south portion of the East Nyainqentanglha Range (IIAB1 d) and south slopes of the East Himalayas Mountains (0A1 b), which are characterized by an annual precipitation exceeding 400 mm, and the vegetation types on the acidic–neutral soils are dominated by coniferous and broad-leaved forests. These soils are favorable for the accumulation of humic acids^56^, which in turn increases their concentrations in flowing water, thereby lowering the pH (see Table 1). Owing to high precipitation (mainly between May–October), moisture retention may occur during drying of grains after harvest, and this promotes contamination by mycotoxin-producing fungi^57,58^. This is a probable origin of the KBD, although the process and mechanism require additional investigations.

### Human activity

The major ion composition of water can be affected many human activities^40^. In the Longzi County, which covers an area of approximately 10,566 km^2^, most of its 33,570 inhabitants^59^ reside in river valleys, flood plains, and along roadways. The economy in this county is essentially rudimentary, with its 2019 regional Gross Domestic Product (GDP) of approximately 1.283 billion yuan indicating a rural per capita disposable income of almost 13,450 yuan. The urbanization rate remains low, and arable, water-occupied, and construction lands account for just 2.28% of the total area. Vast mountainous areas are uninhabited, and serve for few seasonal pastures, thereby highlighting minor human activities. Biogenic components, such as nitrogen- and phosphorus-containing compounds in natural waters commonly reflect the impact of anthropogenic activities^6^. In addition to the major and trace elements composition of the surface waters determined, nitrite (NO_2_^−^) and phosphate (PO_4_^3−^) were measured, and the results are presented in Supplementary Table S2. Evidently, the waters samples are low in NO_2_^−^ and PO_4_^3−^, which indicate human activities minimally impact hydrochemical characteristics of surface waters in the Longzi County. Therefore, in this county, these characteristics are principally controlled by the geological and eco-geographical conditions.,

## Conclusions

In the present study, the pH, TH, and TDS values of surface waters in the KBD endemic areas were characteristically lower than those for the non-KBD endemic areas. In the KBD endemic areas, the waters exhibited dominantly of the Ca–HCO_3_ hydrochemical facies, with subordinate Ca–Mg–HCO_3_ facies, whereas in the non-KBD endemic areas, the Ca–Mg–SO_4_–HCO_3_ facies was prevalent relative to the Ca–Mg–HCO_3_–SO_4_ facies. The hydrochemical characteristics of surface waters in the region displayed correlations with the KBD epidemic. The low salinity waters probably accounted for deficiencies in ETEs, such as Ni, Co, Fe, Se, Zn, Mo, and I in the KBD endemic areas, and PETs, such as As were significantly higher in these areas than in the non-KBD endemic areas. Long-term consumption of low salinity waters deficient in ETEs and containing high PTEs by humans is a likely factor causing KBD in the Longzi County. Environmental factors such as the geology and lithology may produce biogeochemical imbalance, which are the mainly cause for the KBD epidemic. Owing to the complex etiology of the KBD, other environmental impact factors also probably contributed, such as geomorphic, vegetation types and local climatic conditions may have significant impact on food fungi toxin poisoning and water organic compound poisoning.

## Methods

### Study area

**Figure 8:**
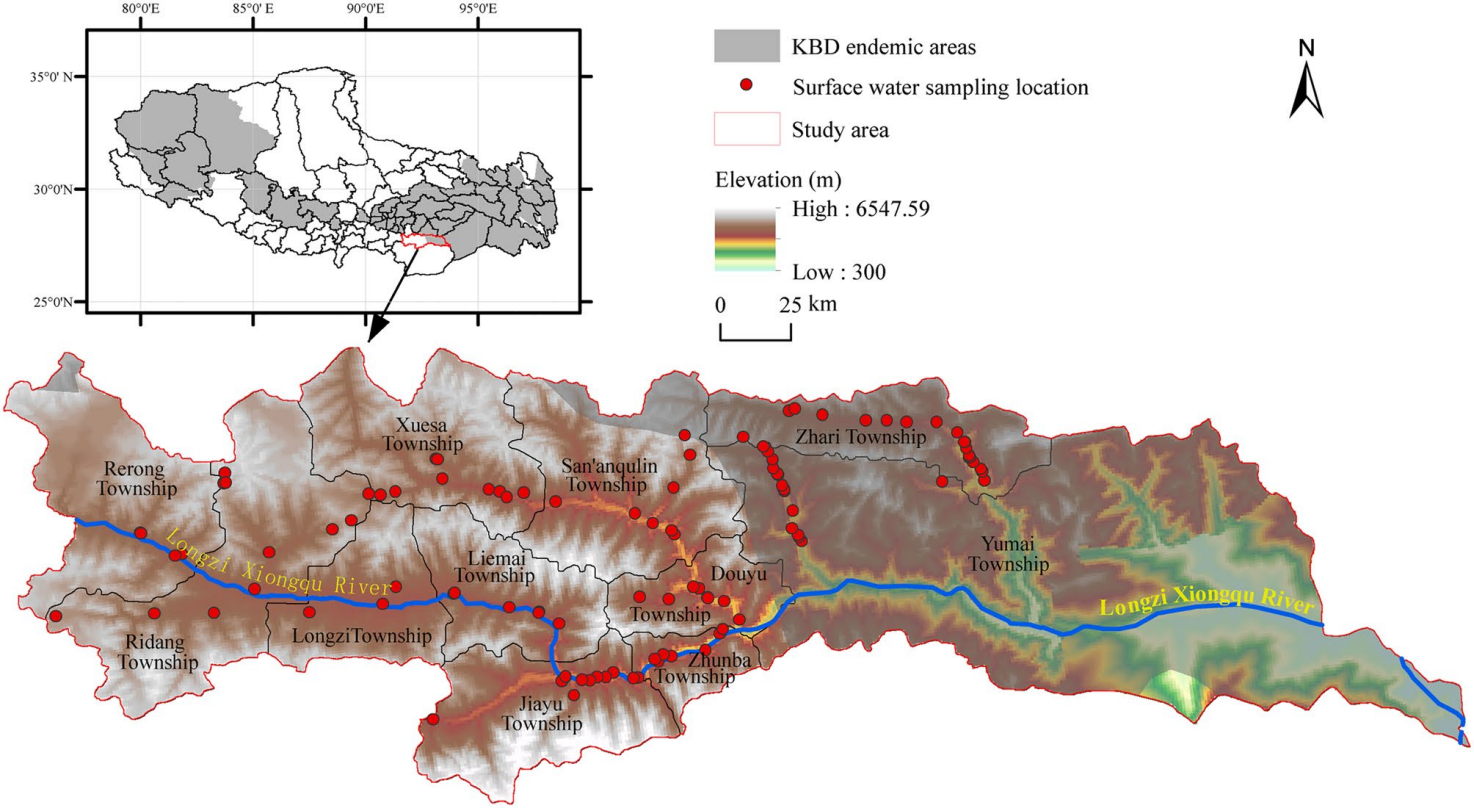
Sampling locations and the KBD endemic areas in the Longzi County (The KBD endemic areas distribution was adapted from Dinh et al.^28^; this figure was created with ArcGIS 10.8, URL: https://www.esri.com/en-us/arcgis/products/index).

The Longzi County (91° 53′–93° 06′ E and 28° 07′–28° 52′ N) is located between the south of the Tibet and the north foot of the east Himalayas, which is characterized by low human activities, involves nine townships unaffected and two affected by the KBD (Fig. 8). This county is in the Himalayan Tectonostratigraphic Zone, includes both Phanerozoic and Precambrian strata. The climate is complex and diverse, with an annual average temperature of approximately 5.5 °C and an annual precipitation between 200 and 600 mm. The topography involves deep valleys and mountains ranging between 300 and 6548 m above sea level. Owing to the climatic specificities, the highland barley is the predominant crop, and most farmlands are in middle reaches of the Longzi River, while the rest are scattered in small plots around villages. The Longzi County, which covers an area of approximately 10,566 km^2^, comprises 11 townships, with a total population of approximately 33,570 Tibetans, Han, and Luoba natives. Grassland, forest, and unused land correspondingly represent 40.65%, 33.87%, and 23.20% of the area, while crop, water-occupied, and construction lands together account for just 2.28%.

### Sampling and analysis

A total of 104 water samples were collected form 11 towns in August 2019 and August 2020 (Fig. 8). In the present study, samples are referred as surface water samples because most were collected from rivers and mountain springs. Although some samples were collected from taps, these originated from mountain streams without any additive. Procedures in the Standard Examination Methods for Drinking Water^60^ were rigorously followed during sampling. Samples were collected from a few centimeters beneath the water surface using polyethylene bottles at each site. To prevent contamination, the bottles were rinsed thrice with the water before the collection of each sample. The bottles were wrapped with a parafilm strip on each cap to prevent leakage.

A portable Global Positioning System (GPS) device (model: 530HCx; produced by Garmin; Taiwan, China) were used to recorded the coordinates of sample locations. The pH, electrical conductivity (EC), ORP and temperature (T) were measured correspondingly using a pH meter (model: SX–620; produced by Sanxing; Shangai, China; with a limit of detection (LOD) of 0.01), an EC tester (model: SX–650; produced by Sanxing; Shanghai, China; with an LOD of 0.1 μS cm^−1^) and an ORP tester (model: SX–630; produced by Sanxing; Shanghai, China; with an LOD of 1 mV). TDS values were obtained from the instrument through automatic conversion of Ec values, while the TH was obtained from the sum of magnesium and calcium concentrations. All water samples were collected and stored according to the Standard Examination Methods for Drinking Water (GB/T 5750-2006)^60^. The analysis of the elements in the water samples were conducted in the Analytical and Testing Center of the Institute of Geographical Sciences and Natural Resources Research (IGSNRR) in Beijing. Concentrations of major cations including Na^+^, Mg^2+^, K^+^, Ca^2+^, etc. were determined using an Inductively Coupled Plasma Optical Emission Spectrometer (ICP–OES; model: Optima 5300 DV; manufactured by PerkinElmer; Massachusetts State, USA; with an LOD of 0.001 mg L^−1^). Trace elements including Al, Si, V, Mn, Fe, Co, Ni, Cu, Zn, As, Se, Mo, I, etc. were analyzed using an Inductively Coupled Plasma Mass Spectrometry (ICP–MS; model: DRC-e; manufactured by PerkinElmer; Massachusetts State, USA; with an LOD of 0.001 µg L^−1^). The concentrations of HCO_3_^−^ and CO_3_^2−^ were determined through alkali titration, while those for Cl^−^, NO_2_^−^, SO_4_^2−^, and PO_4_^3−^ were measured using an Ion Chromatograph (IC; model: ICS–900; manufactured by Thermo Fisher Scientific; Massachusetts State, USA; with an LOD of 0.001 mg L^−1^). During the analyses, quality assurance was achieved using certified external standard solutions and duplicate measurements (a duplicate sample was run after every 10 samples).

### Statistical analysis

The K–S test was used to assess the data distribution, while differences in trace element concentrations between the non-KBD and KBD endemic areas were evaluated using the K–W test^61^. Relationships between trace elements were assessed using correlation analysis, while PCA was used to investigate probable sources of trace elements. Further, the Kaiser–Meyer–Olkin test and Bartlett’s test of sphericity were utilized to ensure that the data was suitable for PCA^62^. Preceding the PCA, the dataset was subjected to the Z-Score Normalization, to eliminate differences associated with the magnitudes of the original variables. Based on the PCA of the normalized dataset, PCs were extracted and those with eigenvalues > 1 were retained^62^. All data were recorded using Microsoft Excel 2019, while statistical analyses were conducted using the SPSS 25.0 software (developed by IBM; Illinois State, USA). The AquaChem 9.0 software (developed by waterloo hydrogeologic; Ontario, Canada) was used to calculate hydrochemical characteristics and plot Piper diagram of water samples. The ArcGIS 10.8 software (developed by ESRI; California State, USA) served for the creation of spatial maps of ion concentrations, while line/scatter plots were produced using the Origin 2021 software (developed by OriginLab; Massachusetts State, USA).

## Supplementary Information

Supplementary Information.
