# Supplementary material for: Hydrochemical characteristics of surface waters and their relationships to the Kashin–Beck Disease in Longzi County, Tibet
Source: Sci Rep. 2022 May 12;12:7819. doi: 10.1038/s41598-022-11463-7 (PMC9098842; doi:10.1038/s41598-022-11463-7)
Supplement: Supplementary file 1 — Supplementary Information. [file 41598_2022_11463_MOESM1_ESM.docx]

**Supplementary Information**

**Hydrochemical characteristics of surface waters and their relationships to the** **Kashin****–Beck Disease in Longzi County, Tibet**


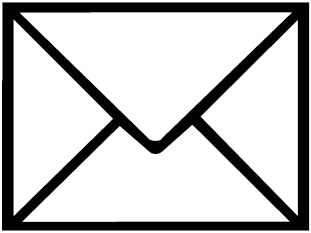

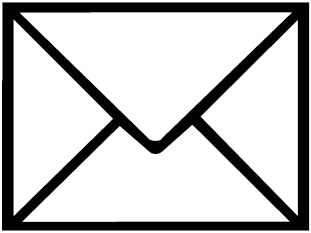
Xinjie Zha^1,#^, Yuan Tian^2,#^ , Jianyu Xiao^2,3^ & Chengqun Yu^2^


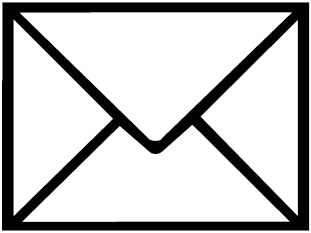
^1^Xi'an University of Finance and Economics, Xi'an, 710100, China. ^2^Key Laboratory of Ecosystem Network Observation and Modelling, Institute of Geographic Sciences and Natural Resources Research, Chinese Academy of Sciences, Beijing, 100101, China. ^3^University of Chinese Academy of Sciences, Beijing 100049, China. email: [tiany.16b@igsnrr.ac.cn](mailto:tiany.16b@igsnrr.ac.cn); [yucq@igsnrr.ac.cn](mailto:yucq@igsnrr.ac.cn). ^#^ These authors contributed equally to this work.

**Table S1.** Eigenvalues, variance, and communalities for trace elements in surface waters from the KBD and non-KBD areas in Longzi County.

| Variable | Non-KBD | | | | | | KBD | | | | | | |
| --- | --- | --- | --- | --- | --- | --- | --- | --- | --- | --- | --- | --- | --- |
|  | PC1 | PC2 | PC3 | PC4 | PC5 | Communalities | PC1 | PC2 | PC3 | PC4 | PC5 | PC6 | Communalities |
| Al | 0.82 | -0.01 | -0.01 | -0.34 | 0.15 | 0.81 | 0.03 | -0.06 | 0.80 | 0.29 | -0.17 | -0.25 | 0.82 |
| Si | -0.16 | 0.22 | 0.21 | 0.86 | 0.01 | 0.86 | 0.87 | -0.19 | 0.03 | -0.11 | -0.16 | 0.05 | 0.84 |
| V | -0.31 | -0.12 | 0.54 | 0.01 | -0.32 | 0.51 | 0.02 | -0.13 | 0.05 | 0.15 | 0.87 | 0.23 | 0.85 |
| Mn | 0.84 | -0.09 | 0.09 | -0.23 | -0.05 | 0.77 | -0.04 | 0.00 | -0.01 | 0.87 | 0.18 | -0.14 | 0.81 |
| Fe | 0.58 | -0.61 | -0.25 | 0.21 | -0.09 | 0.82 | -0.16 | 0.81 | -0.02 | -0.10 | 0.41 | -0.22 | 0.90 |
| Co | 0.74 | -0.04 | -0.04 | 0.56 | -0.04 | 0.87 | 0.91 | 0.05 | 0.04 | 0.06 | 0.03 | -0.05 | 0.84 |
| Ni | 0.87 | -0.03 | -0.12 | 0.12 | -0.03 | 0.79 | 0.44 | 0.63 | -0.07 | 0.11 | 0.44 | -0.09 | 0.82 |
| Cu | 0.01 | 0.20 | 0.78 | 0.12 | 0.16 | 0.68 | 0.13 | -0.02 | 0.26 | 0.75 | -0.03 | 0.30 | 0.74 |
| Zn | 0.03 | 0.02 | 0.52 | -0.20 | -0.34 | 0.42 | 0.84 | -0.04 | -0.07 | 0.11 | 0.15 | 0.03 | 0.75 |
| As | -0.03 | 0.84 | 0.06 | 0.12 | 0.24 | 0.78 | 0.01 | 0.05 | -0.04 | 0.05 | 0.17 | 0.91 | 0.86 |
| Se | -0.02 | 0.80 | -0.18 | 0.14 | -0.23 | 0.74 | -0.02 | 0.86 | 0.04 | -0.02 | -0.20 | 0.03 | 0.78 |
| Mo | -0.02 | 0.03 | 0.03 | -0.03 | 0.89 | 0.80 | -0.15 | 0.85 | -0.01 | 0.03 | -0.26 | 0.26 | 0.87 |
| I | 0.01 | -0.22 | 0.72 | 0.27 | 0.18 | 0.67 | -0.05 | 0.05 | 0.91 | -0.03 | 0.17 | 0.15 | 0.88 |
| Eigenvalues | 3.43 | 1.89 | 1.83 | 1.23 | 1.14 |  | 2.76 | 2.50 | 1.90 | 1.48 | 1.10 | 1.01 |  |
| Variance (%) | 26.40 | 14.50 | 14.04 | 9.49 | 8.80 |  | 21.21 | 19.20 | 14.64 | 11.38 | 8.43 | 7.79 |  |
| Cumulative (%) | 26.40 | 40.90 | 54.93 | 64.43 | 73.23 |  | 21.21 | 40.41 | 55.05 | 66.43 | 74.86 | 82.65 |  |

**Note:** KMO result is >0.5 and the significance of Bartlett’s test of sphericity is <0.001

| County | Longzi | Ridang | Liemai | Rerong | Sananqulin | Zhunba | Xuesa | Zhari | Yumai | Jiayu | Douyu |
| --- | --- | --- | --- | --- | --- | --- | --- | --- | --- | --- | --- |
| NO_2_^–^ | 0.026 | 0.006 | 0.000 | 0.000 | 0.032 | 0.000 | 0.000 | 0.000 | 0.000 | 0.000 | 0.026 |
| PO_4_^3–^ | 0.000 | 0.000 | 0.000 | 0.000 | 0.000 | 0.000 | 0.000 | 0.000 | 0.000 | 0.000 | 0.000 |

**Table S2** Concentrations of anthropogenic input compounds for surface water in the Longzi County, expressed in mg L^–1^.

**Note:** Values of 0.000 means the concentration of the compounds is not detected or is below the LOD.
